# Precursors for Atmospheric Plasma‐Enhanced Sintering: Low‐Temperature Inkjet Printing of Conductive Copper

**DOI:** 10.1002/open.201800131

**Published:** 2018-08-31

**Authors:** Caroline E. Knapp, Elizabeth A. Metcalf, Shreya Mrig, Clara Sanchez‐Perez, Samuel. P. Douglas, Patrick Choquet, Nicolas D. Boscher

**Affiliations:** ^1^ Department of Chemistry University College London 20 Gordon Street London WC1H 0AJ UK; ^2^ Department of Materials Research and Technology Luxembourg Institute of Science and Technology 5 Avenue des Hauts-Fourneaux Esch/Alzette 4362 Luxembourg

**Keywords:** atmospheric plasma, conductive copper, copper precursors, inkjet printing, plasma-assisted inkjet printing

## Abstract

Bidentate diamine and amino‐alcohol ligands have been used to form solid, water‐soluble, and air‐stable monomeric copper complexes of the type [Cu(NH_2_CH_2_CH(R)Y)_2_(NO_3_)_2_] (**1**, R=H, Y=NH_2_; **2**, R=H, Y=OH; **3**, R=Me, Y=OH). The complexes were characterized by elemental analysis, mass spectrometry, infrared spectroscopy, thermal gravimetric analysis, and single‐crystal X‐ray diffraction. Irrespective of their decomposition temperature, precursors **1**–**3** yield highly conductive copper features [1.5×10^−6^ Ω m (±5×10^−7^ Ω m)] upon atmospheric‐pressure plasma‐enhanced sintering.

## Graphical abstract

**Hot off the press**: Bidentate diamine and amino‐alcohol ligands are used to form solid, water‐soluble, and air‐stable monomeric copper complexes. Irrespective of their decomposition temperature, the precursors each yield highly conductive copper features upon atmospheric‐pressure plasma‐enhanced sintering.

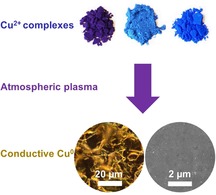

## 1 Introduction

The market for printed electronics has seen steady growth over the last decade, particularly as lower processing temperatures have facilitated a move to low‐cost flexible materials.^1^ High‐quality and, therefore, low‐electrical‐resistance printed features that can replace electrical wiring in a range of devices, particularly the shunting lines in organic light‐emitting diodes (OLEDs), interconnects for photovoltaics, and the antennae in radio‐frequency identification (RFID) devices, are in urgent need. Inkjet printing of metallic features is fairly common place industrially; typically nanoparticle (NP)‐based formulations are used.^2^ These “inks” consist of metal nanoparticles dispersed in a solvent that also contains stabilizing agents and other substances to control the viscosity, consistency, and wettability of the ink.^3^ Sophisticated conditions are often required for their deposition onto substrates, as well as high temperatures for sintering (>150 °C), which raises the cost and limits the range of suitable substrates.^4^, ^5^

Metal‐organic decomposition (MOD) inks, as an alternative, provide potential for higher economic feasibility^6^ and more widespread use. Owing to its lower cost and tendency for electromigration, with the passage of high electron current densities,^7^ copper is an attractive alternative to other highly conductive metals, for example, silver. Copper MOD inks typically consist of a copper precursor, that is, a metal ion, oxidized and bound to ligands, such that upon specific treatment (usually heating)^2^, ^8^ the metal center is reduced and the ligands decompose cleanly, leaving conductive metal features. Complexation of copper(II) with different ligands gives rise to intricate decomposition properties, owing to the varied stabilization offered by different covalent and non‐covalent interactions. Strategic variation of the organic ligands affords the ability to tune the precursor properties: the lower its decomposition temperature, the higher the potential for use in conductive inks that can be printed on low‐cost substrates.

Copper(II) nitrate and copper(II) formate have received much attention as viable precursors in both the chemical vapor deposition (CVD) and inkjet printing of metallic copper, despite the latter's propensity to evolve formic acid and, therefore, degrade the ink when mixed with protic additives.^9^ The current state‐of‐the‐art copper(II) formate ink formulations consist of in situ copper(II) formate solutions with co‐complexing agents, usually amines and their derivatives. Yabuki et al. reported the deposition of films of conductive copper through the thermal decomposition of mixtures of copper(II) formate and *n*‐octylamine under a nitrogen (inert) atmosphere.^10^ The film with the lowest resistivity (2×10^−5^ Ω cm) was obtained after heating for 60 min at 140 °C.^11^ Inks have been reported with improved results when a ratio of different amines are used.^12^ The best performing ink contained a blend of amines in a 20 mol % dibutylamine‐80 mol % octylamine mixture. This formulation demonstrated significant improvement (resistivity of 5×10^−6^ Ω cm), although sintering at 140 °C still took 30 min. A copper film of comparable resistivity to this was produced by Kim et al. with a formulation of copper(II) formate and hexylamine.^13^ The resistivity of the film measured 5×10^−6^ Ω cm at a sintering temperature of 200 °C for 2 min. This demonstrates the on‐going compromise that must be made between curing temperature and time.

Farraj et al.^14^ evaluated a range of amino‐hydroxyl complexes as complexing ligands with copper(II) formate. Amino hydroxyl complexes provide several advantages over others, including an improved stability during processing and storage (up to 3 months), a decomposition which occurs at sufficiently low temperatures and good solubility of their copper complexes in glycol ether solvents. Compatibility with glycol ether solvents is advantageous, as they are commonly used as a liquid vehicle in inkjet printing, owing to their use in tuning viscosity of the ink.^14^, ^15^ It was found that use of 2‐amino‐2‐methyl‐1‐propanol (AMP) led to both a decrease in the decomposition temperature and the achievement of the best conductivity for the copper metal produced. It was suggested by Shin et al.^15^ that the presence of the tertiary carbon, a feature unique to AMP compared to the other complexes tested, contributes to the formation of small volatile byproducts. The hindered carbon, which is not present in other amino‐hydroxyl ligands, inhibits the formation of stable carbamate ions, thereby restricting polymerization reactions between carbon dioxide and other organic byproducts.^16^ Shin et al.^15^ conducted a similar investigation using copper(II) formate and AMP whilst also including a co‐complexing agent, octylamine, to increase physical contact between copper particles following sintering and, therefore, improve conductivity of the films produced. It was shown that the addition of both the amine and the amino‐hydroxyl complexes, together, did result in smaller, more densely packed particles; however, the sintering time and temperature (i.e. 30 min, 300 °C) of the complexes reported are high. In most cases, thermal sintering has been used to form conductive copper; however, it should be noted that using UV^17^, ^18^ or microwave^19^ irradiation, an argon ion LASER beam,^20^ low‐pressure plasma,^21^ or pulse electric current^22^ has also previously been reported. In particular, Farraj et al. recently reported the use of an in situ copper(II) formate and AMP ink with low pressure plasma^23^ to form conductive copper features. The plasma‐enhanced sintering procedure was operated in the absence of other external heating and under 0.2 mbar vacuum. The MOD ink needed to be exposed to 160 W plasma for 8 min to yield conductive copper (7×10^−6^ Ω cm).

**Scheme 1:**
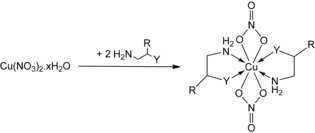
Synthesis of **1**–**3** (**1**: Y=NH_2_, R=H; **2**: Y=OH, R=H; **3**: Y=OH, R=Me).

Despite these investigations into the types of “in situ” ink formulations, which have shown much promise in depositing conductive copper, no notable attempts have been made to design, synthesize, and isolate copper(II) complexes. Isolated copper(II) complexes that can be further formulated into precursor inks to circumnavigate the shelf‐life issues associated with using reactive in situ mixtures. Indeed, the production of solid and stable MOD inks would considerably improve the inkjet printing of conductive features by preventing excessive and detrimental wetting of the substrates, including paper, as well as early decomposition.^24^ From the coordination of bidentate diamine and amino‐alcohol ligands with copper(II) nitrate to produce isolated precursors, this work aims to contribute toward a greater understanding of the relationship between functionality of ligands and resultant ink performance, with the hope that this new knowledge will assist in the selection of more effective directions for the development of ink precursors for the low‐temperature plasma‐enhanced sintering of highly conductive metallic features.^25^ These new isolated copper precursors, which are solid, water‐soluble, and stable under ambient conditions, have been investigated with the atmospheric‐pressure plasma‐assisted inkjet printing that was recently demonstrated as a versatile room‐temperature approach that can be upscaled for the immediate conversion of MOD inks (Scheme 1).^24^

## 2 Results and Discussion

While targeting the preparation of solid and stable copper MOD precursors for the plasma‐assisted inkjet printing of copper, a solution of copper nitrate trihydrate and two equivalents of ethylenediamine in methanol were stirred overnight to form [Cu(NH_2_CH_2_CH_2_NH_2_)_2_(NO_3_)_2_)] (**1**) in solution. After the solution was filtered and left at −5 °C for 1 week, bright violet crystals of a suitable quality for single‐crystal X‐ray diffraction (SCXRD) formed, which confirmed the isolation of **1** consistent with the literature.^26^

**Figure 1:**
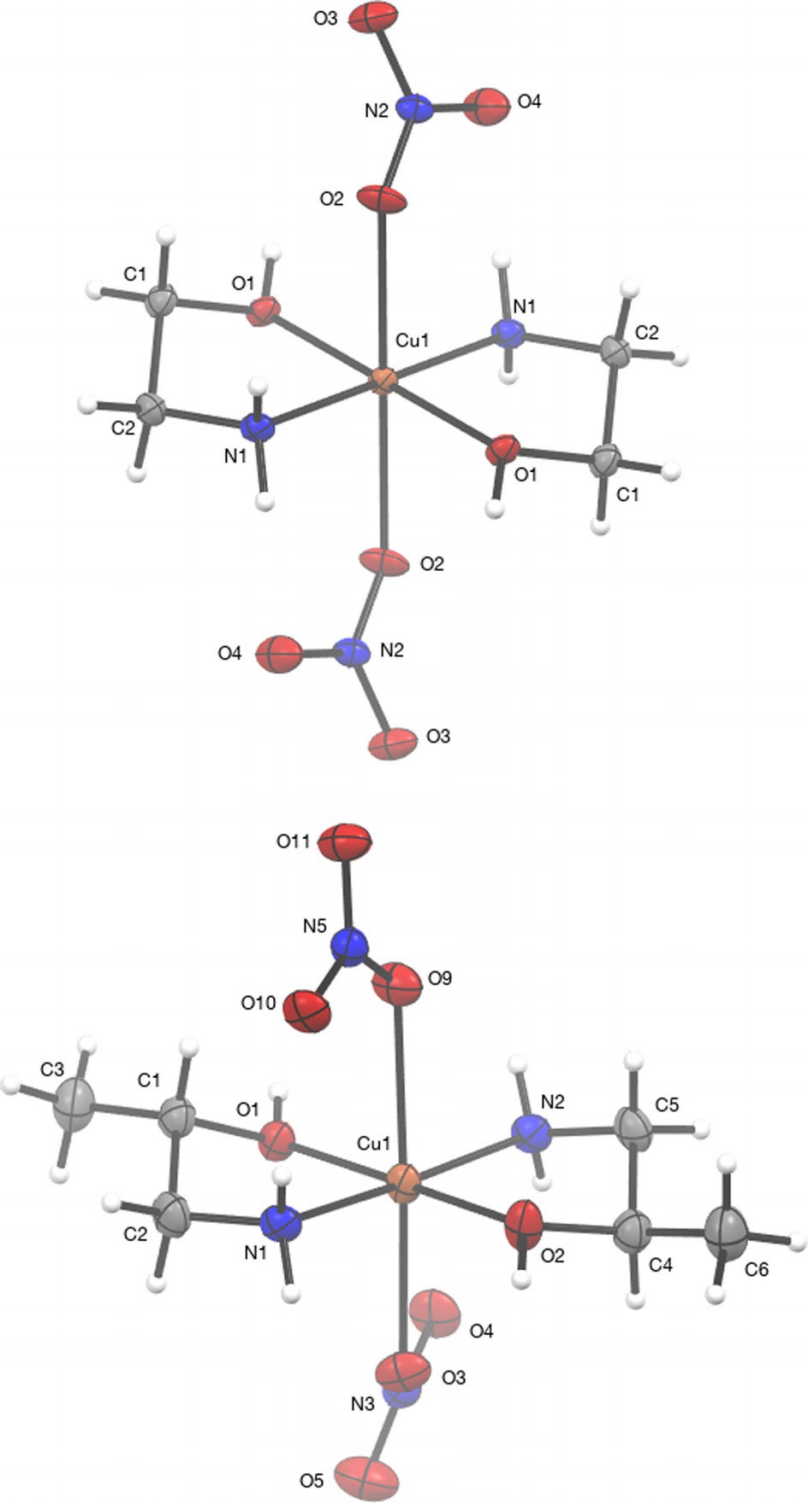
The molecular structure of **2** (top) and **3** (bottom). Thermal ellipsoids drawn at 50 % probability.

For the synthesis of the isostructural complexes **2** and **3**, a solution of copper nitrate trihydrate was mixed with two equivalents of ethanolamine (**1**) or amino‐2‐propanol (A2P) (**2**) in methanol (**1**) or ethanol (**2**), and refluxed overnight. After filtering the solutions, the filtrates were left at −5 °C for 7 days, after which bright blue crystals of [Cu(NH_2_CH_2_CH_2_OH)_2_(NO_3_)_2_)] (**2**) and [Cu(NH_2_CH_2_CH(Me)OH)_2_(NO_3_)_2_)] (**3**) had formed. Isolation of complexes **2** and **3** was confirmed by using elemental analysis (EA), mass spectrometry (MS), and infrared spectroscopy (IR), and recrystallization afforded suitable crystals for SCXRD. The structure of these complexes consists of centrosymmetric units crystallized in a triclinic *P* $\bar{1}$ space group (Figure 1). The copper(II) ion is in a [4+2] environment. The four atoms in the basal plane about copper are its nearest neighbors [the two nitrogen (N1, N1′/N2) and two oxygen atoms (O1, O1′/O2) from the two amino‐alcohol molecules], which act as bidentate coordination ligands.

These are in the equatorial positions with comparable Cu−O bond lengths [1.9828(12) Å (**1**), 1.9917(13) Å (**2**)] and Cu−N bond lengths [1.9745(14) Å (**1**), 1.9801(15) (**2**)]. The axial positions are occupied by oxygen atoms of two nitrate anions at greater distance [2.576 Å for Cu1–O2 (**1**), 2.4232(14) (**2**)]. The short bond angle about the copper center in the five‐membered ring [84.59(5) Å for O1–Cu1–N1 (**1**); 83.52(6) Å for O1–Cu1–N1 (**2**)] is attributed to the geometrical constraints in the coordinated amino‐alcohol ligands. The only significant difference in bond lengths between **2** and **3** is for the oxygen and adjacent carbon atom in the ligand (Table 1). In **3**, these are slightly longer [1.458(2) Å for O1–C1 (**1**); 1.452(2) Å for O2–C4 (**2**)], owing to the presence of the methyl group on these respective carbons. Examination of the bond angles around the central copper in **3** reveal that the CuO_2_N_2_ plane is slightly distorted from planarity; for example, the O1–Cu1–O2 bond angle is 176.69(5)° rather than 180°, as would be expected for a perfectly square planar base. A similar observation can be made on the N1–Cu1–N2 bond angle. The nitrate group with central N3 is closer to the copper than the second nitrate with central N4 (2.435 Å for Cu1–O3; 2.536 Å for Cu1–O6). As it is closer to the complex ion, O5 on the nitrate can form a stronger hydrogen bond with hydrogen atoms on N1, than O8 can with hydrogen atoms on N2. As a result, the rest of the atoms in the central complex are distorted in one direction, making the complex asymmetric. The asymmetry of **3** reduces its stability and, therefore, its decomposition temperature, adding to its potential for use as a copper precursor. Further crystallographic data for complexes **2** and **3** are provided in Table 2.

**Table 1:** Selected bond lengths [Å] and angles [°] for amino‐hydroxyl copper(II) complexes **2** and **3**.

	[Cu(EA)_2_(NO_3_)_2_] (**2**)	[Cu(A2P)_2_(NO_3_)_2_] (**3**)
**Lengths [Å]**		
Cu1–O1	1.9828(12)	1.9917(13)
Cu1–O2	–	1.9864(14)
Cu1–N1	1.9745(14)	1.9801(15)
Cu1–N2	–	1.9782(15)
O1–C1	1.444(2)	1.458(2)
O2–C4	–	1.452(2)
N1–C2	1.481(2)	1.481(3)
N2–C5	–	1.476(2)
C1–C2	1.512(2)	1.506(3)
C4–C5	–	1.510(3)
Cu1–O2	2.576*	–
Cu1–O3	–	2.4232(14)
Cu1–O6	–	2.536^[a]^
**Angles [°]**		
O1′–Cu1–O1	180.0	–
O1–Cu1–O2	–	176.69(5)
N1–Cu1–N1	180.0	–
N1–Cu–N2	–	174.44(6)
O1–Cu1–N1	84.59(5)	83.52(6)
O2–Cu1–N2	–	83.57(6)
O1′–Cu1–N1	95.41(5)	–
O2–Cu1–N1	–	93.34(6)
N2–Cu1–O1	–	99.48(6)
C1–O1–Cu1	110.03(10)	109.51(11)
C4–O2–Cu1	–	110.67(11)
C2–N1–Cu1	108.28(10)	109.24(12)
C5–N2–Cu1	–	108.53(12)

[a] This interaction is not a formal bond, so distance has been calculated using fractional atomic coordinates.

**Table 2:** Crystallographic data for complexes **2** and **3**.

	[Cu(EA)_2_(NO_3_)_2_] (**2**)	[Cu(A2P)_2_(NO_3_)_2_] (**3**)
Crystal system	triclinic	triclinic
Space group	*P* $\bar{1}$	*P* $\bar{1}$
*a* [Å]	6.5391(3)	8.2442(4)
*b* [Å]	6.6288(3)	9.4589(4)
*c* [Å]	7.3234(4)	9.9484(4)
*α* [°]	111.725(5)	103.064(4)
*β* [°]	102.374(5)	101.340(4)
*γ* [°]	98.579(4)	114.074(4)
Volume [Å^3^]	278.76(3)	652.58(6)
*Z*	2	2
*ρ*_calc_ [g cm^−3^]	1.845	1.7189
*Μ* [mm^−1^]	3.215	2.803
*F*(000)	159.0	347.3
Crystal size [mm^3^]	0.1×0.1×0.05	0.5×0.1×0.1
2*θ* range for data collection [°]	13.614–146.332	9.64–147.28
Index ranges	−8 ≤ *h* ≤7, −8 ≤ *k* ≤8, M‐>7 ≤ *l* ≤9	−10 ≤ *h* ≤10, −11 ≤ *k* ≤11, −12 ≤ *l* ≤11
Reflections collected	3263	9415
Independent reflections	1088 [*R*_int_=0.0323, *R*_sigma_=0.0247]	2574 [*R*_int_=0.0291, *R*_sigma_=0.0215]
Data/restraints/parameters	1088/3/82	2574/6/180
Goodness‐of‐fit on *F* ^2^	1.136	1.052
Final *R* indexes [*I*>=2*σ* (*I*)]	*R*1=0.0244, *wR*2=0.0633	*R*1=0.0308, *wR*2=0.0804
Final R indexes [all data]	*R*1=0.0251, *wR*2=0.0636	*R*1=0.0335, *wR*2=0.0827
Largest diff. peak/hole [e Å^−3^]	0.35/−0.26	0.44/−0.30

Interestingly, attempts to use AMP as a ligand to isolate an isostructural octahedrally coordinated complex of the type [Cu(NO_3_)_2_(AMP)_2_] to use as a precursor were not successful; instead, only the less reactive compound [Cu(OCH_2_C(CH_3_)_2_NH_2_)_2_⋅H_2_O] could be isolated, which has been previously reported.^27^

When considering the suitability of these complexes as precursors to copper metal, their structure is most important, as metal–ligand bond length and, therefore, strength is driving the decomposition temperature. Complexes **1**–**3** all maintain their copper‐to‐nitrate ligand bond and the diamine/amino‐alcohol ligands are coordinated, exhibiting a Jahn–Teller distorted octahedral geometry; the coordination length of the nitrate groups through the oxygen atoms is slightly longer than that of the nitrogen atoms in the diamine bidentate ligands. The diamine molecules are in the *gauche* configuration; therefore, the CH_2_ groups in the ligand are asymmetrical about the CuN_4_ plane. Overall, the high copper weight % of these complexes (**1**: 20.65 %; **2**: 20.52 %; **3**: 18.81 %) suggests that they may be good copper precursors. Additionally, the presence of the counter‐ion nitrate groups suggests that the interactions with the amine groups may be weaker and, therefore, the decomposition temperature of these complexes should be low.

### 2.1 Thermal Studies

**Figure 2:**
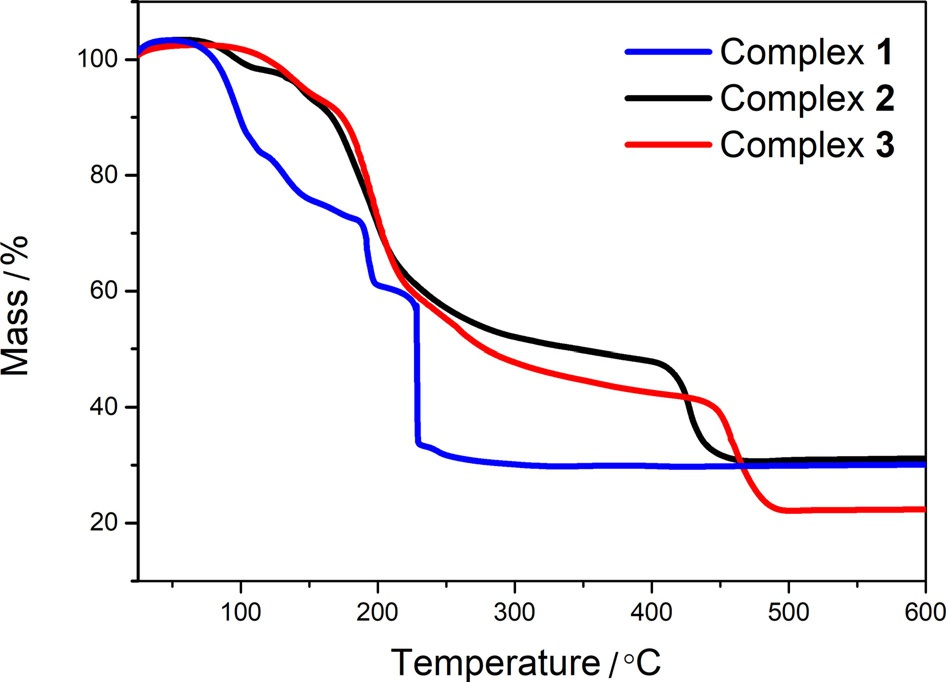
TGA data for complexes **1**–**3**.

The thermogravimetric analysis (TGA) of complexes **1**–**3** provides useful information concerning the decomposition temperature and profile of each complex that can be used to predict the thermal sintering behavior of the inks (Figure 2). Complex **1** begins to lose mass at approximately 75 °C followed by three more stages, likely owing to the dissociation of the ligands; decomposition to 30 % of starting mass is complete by 230 °C. The change from a diamine ligand to amino‐alcohol is evident when comparing the TGA data from complex **1** with complexes **2** and **3**, which are very similar to each other. For **2** and **3**, the onset of decomposition begins at approximately 90 and 100 °C, respectively. In each case, the decomposition proceeds in two steps, with the first decomposition stage yielding a 30 % loss of the starting mass and the second one completed at 450 °C (70 %) for **2** and 495 °C for **3** (80 %).

### 2.2 Printing and Plasma‐Enhanced Sintering

As evidenced by TGA, the three complexes reported in this work decompose at fairly high temperatures, that is, 230 to 495 °C. Consequently, the thermal sintering of inks formulated from the studied complexes implies the use of temperature in the same range to yield metallic copper. These temperatures prevent the use of numerous substrates, for example, polymers and paper. As reported in several works, the temperature limitation raised by the thermal sintering approach can be circumvented by using a plasma to enhance the sintering.^23^, ^24^ However, plasma‐enhanced sintering does not resolve all of the complications, such as wetting and early decomposition. Recent studies on the atmospheric‐pressure plasma‐assisted inkjet printing of highly conductive silver features on paper have notably highlighted the wetting and stability issues encountered when working on porous substrates at ambient conditions.^24^ Thus, solid and air‐stable MOD complexes, which can be made into aqueous inks, are highly desirable for simple room‐temperature plasma‐assisted inkjet printing of functional devices. Conveniently, the three copper complexes reported above are all solid, water‐soluble, and stable under the laboratory atmosphere, making them suitable candidates for inkjet printing and atmospheric plasma‐enhanced sintering. Complexes **1**–**3** were made up to 0.14 m aqueous solutions to formulate inks **1**–**3**, and were subsequently used to print tracks onto glass substrates. Following their deposition, the films of precursor inks were plasma‐enhanced sintered to reduce the copper ions and yield a pure metal film. An atmospheric‐pressure dielectric barrier discharge (AP‐DBD) configuration operated at atmospheric pressure and supplied with an argon‐hydrogen mixture was selected, owing to its suitability for the treatment of large surfaces.^28^ A 97.5:2.5 argon–hydrogen plasma gas composition was selected to maximize the concentration of reactive hydrogen species^29^ that favor the low‐temperature reduction of MOD ink^30^ and promote a non‐thermal desorption of the ligands.^31^ In contrast to our previous work, where the plasma‐assisted inkjet printing of silver required numerous fast and short inkjet printing/plasma‐enhanced sintering cycles (ca. 2 s) to prevent the decomposition of the silver MOD ink to silver oxide, the present work involves only three cycles. These cycles consist of a fast inkjet printing step followed by a plasma‐enhanced sintering step that was set to 40 min to ensure the full conversion of the inks and to produce thin films with decent thicknesses (2–4 μm) for surface analysis.

Conductive copper [1.5×10^−6^ Ω m (±5×10^−7^ Ω m)], with a resistivity 100 times higher than bulk copper (1.68×10^−8^ Ω m) was achieved for the three ink solutions and the same deposition procedure. X‐ray photoelectron spectroscopy (XPS) analysis of the copper coatings revealed a high relative weight concentration of Cu, that is, 79.3, 75.2, and 69.7 wt % for conductive copper coatings elaborated from inks **1** to **3**, respectively. It is interesting to note that, despite a decomposition temperature close to that of complex **3** (i.e. **2**: 450 °C and **3**: 495 °C), complex **2** yields a significantly higher copper content of the produced coating. Quite strikingly, this copper content (75.2 wt %) is very close to that of the coating elaborated from complex **1**, which possesses a much lower decomposition temperature (i.e. **1**: 230 °C vs. **2**: 450 °C), but shares a similar initial copper content in the complexes (**1**: 20.65 %; **2**: 20.52 %; **3**: 18.81 %). Thus, when designing new precursors for the atmospheric‐pressure plasma‐assisted inkjet printing of metallic coatings, one may not exclusively target the lowest decomposition temperature, but the use of smaller ligands that can desorb more readily.

**Figure 3:**
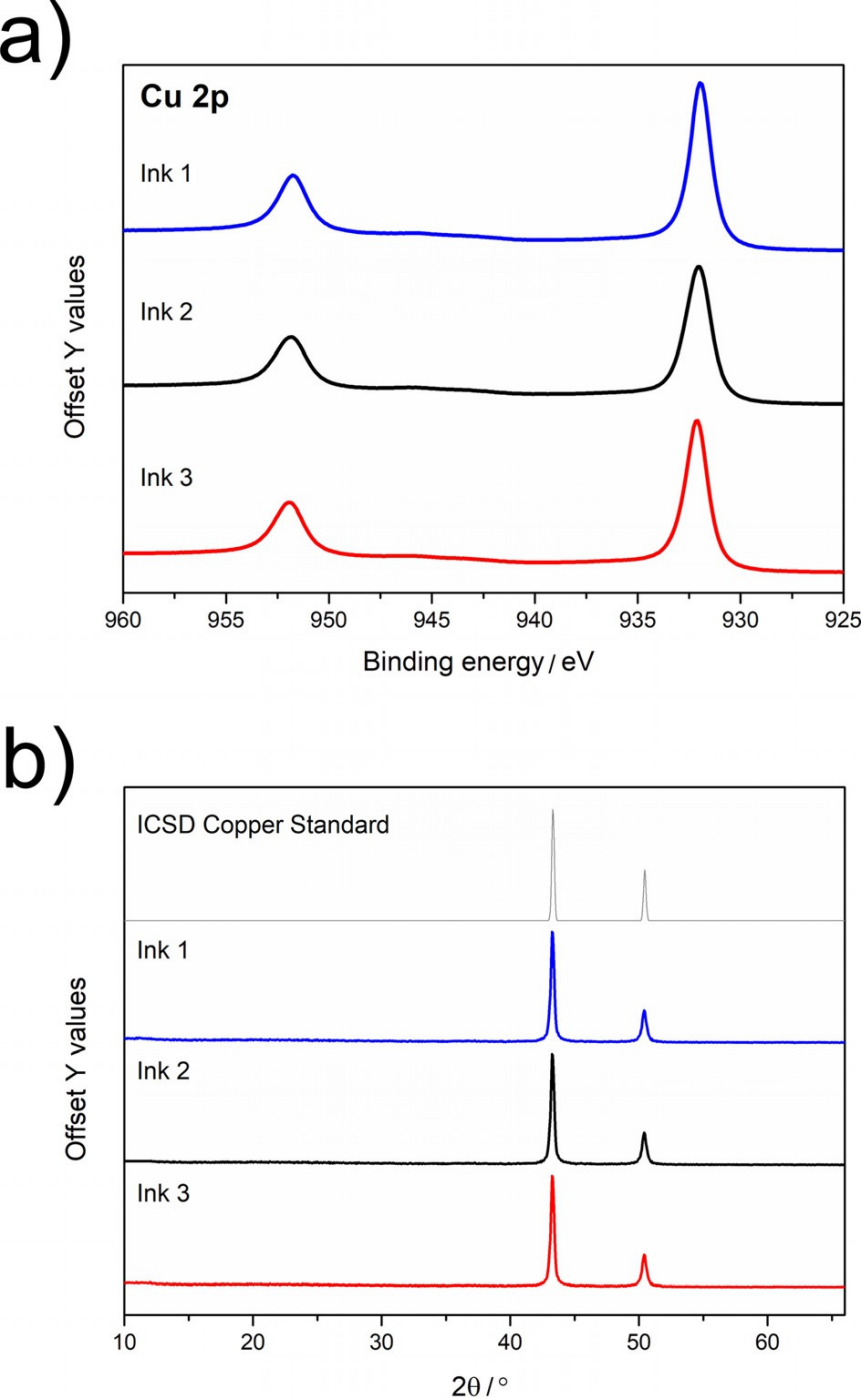
XPS of Cu 2p transitions (top), all deposited using print/plasma cycles of complexes **1**–**3**, XRD patterns (bottom) for ICSD standard and deposited copper.

For all of the prepared coatings, and irrespective of the MOD ink, XPS analysis revealed two peaks at 951.78 and 931.98 eV, corresponding to the 2p_1/2_ and 2p_3/2_ peaks of metallic Cu, respectively (Figure 3 a). The positions of the Cu 2p spin–orbit components, separated by approximately 20 eV, is consistent with the formation of metallic copper. X‐ray diffraction (XRD) analysis (Figure 3 b) of the copper deposited from inks **1**–**3** confirms the formation of copper, with no evidence of impurities (copper oxides or unconverted precursor).

**Figure 4:**
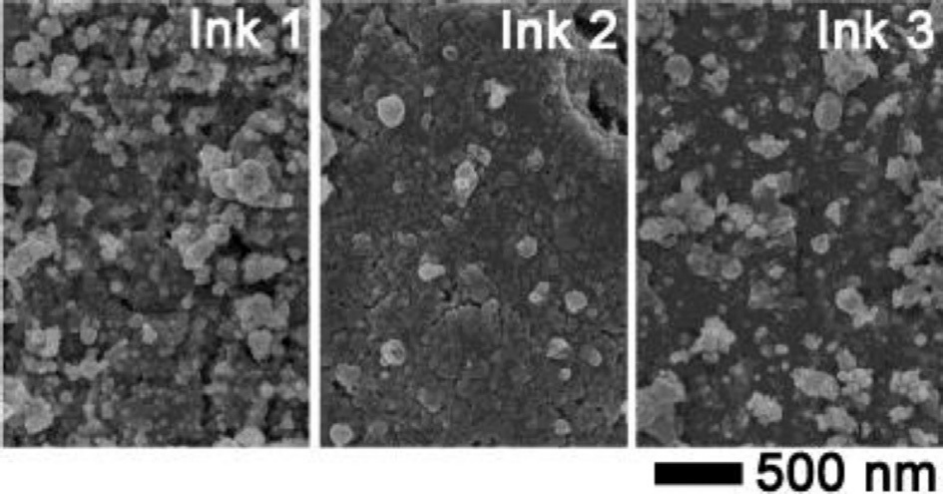
SEM images of films deposited from **1**–**3**.

Scanning electron microscopy (SEM) images reveal the rather dense morphology of the copper coatings composed of particles ranging from 50 to 200 nm (Figure 4). It is during the sintering process that the metal particles form connections between themselves, creating a continuous percolating network throughout the film.^32^ The low resistance of the copper coatings coupled to their rather dense morphology compared to other MOD ink‐based coatings underline the suitability of the synthesized complexes for the proposed plasma approach. The use of plasma rather than the conventional thermal heating methods highlights the potential future for targeted precursor design and synthesis, resulting in low‐temperature processing. Indeed, the AP‐DBD configuration employed in the present study produces non‐thermal plasma that induces a reduced increase in the substrate temperature. Even for long processing times, the temperature did not exceed 70 °C. On the other hand, AP‐DBD produces a wide variety of reactive species, that is, electrons, ions, radicals, metastables, and photons, which all possess a significant energy that can easily break chemicals bonds.^33^, ^34^ As an example, in AP‐DBD, the mean electron energy is in the range of 1–10 eV. In the present work, precursors **1**–**3** undergo reduction to metallic copper when they are exposed to an argon–hydrogen atmospheric plasma. This result can be partly attributed to the longer and, therefore, weaker bond between the copper(II) center and its ligands. In addition, the molecules’ asymmetry, which causes dipole moments within the complex, induces stronger intermolecular interactions^35^ that increase the oligomerization of the complexes, resulting in a reduced volatility and improving their suitability as precursors.^36^

## 3 Conclusions

Three solid, water‐soluble, and air‐stable copper coordination complexes have been synthesized, characterized, and successfully investigated for the low‐temperature deposition of metallic copper layers by atmospheric‐pressure plasma‐assisted inkjet printing. In particular, the use of isolated copper coordination complexes, whose crystallographic data are reported for the first time, provided a convenient approach to prevent the early decomposition of the inks. More importantly, the plasma‐assisted inkjet printing of these designed precursors **1**–**3** has been shown to be an effective alternative to thermal sintering. Irrespective of the decomposition temperature of complexes **1** to **3** (230 to 495 °C), it yields highly conductive copper features at temperatures lower than 70 °C. This work could be further extended in the future to deposit highly conductive copper features onto a range of different substrates, including paper and plastics, such as previously demonstrated for the plasma‐assisted inkjet printing of silver.^24^

## Experimental Section

Crystallographic/refinement data for complexes **2** and **3** (https://www.ccdc.cam.ac.uk/services/structures?id=doi:10.1002/open.2018001311864245–1864246) can be found online.^37^ Mass spectrometry was performed on a Thermo Finnigan MAT900 XP operating in electron impact (EI) and chemical ionization (CI) modes. Single‐crystal X‐ray diffraction datasets were collected on a SuperNova (dual source) Atlas diffractometer, using either monochromated Cu *K*_α_ radiation (*λ*=1.54184 Å) or monochromated Mo *K*_α_ (*λ*=0.71073 Å).

### General Procedures

All reagents and solvents were procured from Sigma Aldrich and used as received with no further purification. All IR spectra were recorded by using a Shimadzu FTIR‐8200 spectrometer, operating in the region of 4000–400 cm^−1^. EA was carried out by using an elemental analyzer (CE−440) (Exeter Analytical Inc.). The X‐ray single‐crystal diffraction (SCD) experiments were carried out by using an Atlas diffractometer. A suitable crystal was selected and mounted on a nylon loop on a SuperNova, Dual, Cu/ Mo. The crystal was kept at 150 K during data collection. Using Olex2,^38^ the structure was solved with the Superflip^39^ (**2**, **3**) structure solution program, using Charge Flipping and refined with the olex2.refine^40^ (**2**) or ShelXL^41^ (**3**) refinement package with Gaussian–Newton or Least Squares minimization, respectively. The instrument used for simultaneous thermal analysis was a Netzsch STA 449C. All measurements were carried out with the precursor sample in an aluminum crucible. The data were recorded from room temperature (20 °C) to 600 °C. XRD patterns were recorded with a Bruker D8 Discover X‐ray diffractometer by using monochromatic Cu *K*_α1_ and Cu *K*_α2_ radiation of wavelengths 1.54056 and 1.54439 Å, respectively, emitted in an intensity ratio of 2:1 with a voltage of 40 kV and a current of 40 mA. The diffraction pattern was taken over 2*θ*=10–66. SEM was performed with a Philips XL30 FEG instrument operating in plan mode with an electron‐beam accelerating energy of 30 kV and an instrument magnification of 50000×. XPS was performed with a Thermo Scientific K‐Alpha XPS system by using monochromatic Al *K*_α_ radiation at a 1486.6 eV X‐ray source. CasaXPS software was used to analyze the data with binding energies referenced to an adventitious C 1s peak at 284.8 eV.

### Synthesis of Bisethylenediaminecopper(II) Nitrate (1)

Cu(NO_3_)_2_.3 H_2_O (0.25 g, 1.03 mmol) was dissolved in methanol (MeOH) (2 mL), and then ethylenediamine (en) (0.124 g, 2.06 mmol) dissolved in MeOH was added. The solution was covered and stirred overnight at room temperature. Following this, the solution was filtered by using gravity, separating a blue solute from a purple precipitate. The solute was left at −5 °C to allow crystals to grow. After 1 week in the freezer, violet crystals were found. Experimental analysis for [Cu(en)_2_(NO_3_)_2_]: Yield: 79 %; Elemental analysis found: C 15.20, H 5.27, N 25.82, calculated for [Cu(en)_2_(NO_3_)_2_] C 15.61, H 2.60, N 27.32; FTIR (cm^−1^): 3324 (N‐H), 3270 (N‐H), 3245 (N‐H), 3157 (N‐H), 2954 (C−H), 2941 (C−H), 2884 (C−H), 1602 (N‐H bend); Mass spectrometry: ES‐LRMS [M‐NO_3_]^+^ 245.1 (100 %).

### Synthesis of Bis(ethanolamine)copper(II) Nitrate (2)

Cu(NO_3_)_2_.3H_2_O (6 g, 24.8 mmol) was dissolved in MeOH (30 mL), and then ethanolamine (3.06 g, 89.9 mmol) dissolved in MeOH (45 mL) was added. Upon addition of the ethanolamine, the solution turned from pale blue to dark blue. The mixture was refluxed and stirred overnight. After 21 h, the mixture was filtered by using gravity to remove a blue precipitate and some solvent was removed from the clear blue solution with a rotary evaporator before it was left at −5 °C to allow crystals to grow. After 7 days in the freezer, dark blue crystals were found. Experimental analysis for [Cu(EA)_2_(NO_3_)_2_]: Yield: 1.40 g, 84 %; Elemental analysis found: C 15.6, H 4.60, N 17.96, calculated for [Cu(EA)_2_(NO_3_)_2_] C 15.5, H 4.52, N 18.09; FTIR (cm^−1^): 3314 (N‐H), 3263 (N‐H), 2975 (C−H), 2954 (C−H), 2902 (C−H), 2861 (C−H), 2668 (C−H), 1570 (N‐H bend); Mass spectrometry: ES‐LRMS [M‐2NO_3_‐H]^+^ 184.0 (100 %).

### Synthesis of Bis(amino‐2‐propanol)copper (II) Nitrate (3)

Cu(NO_3_)_2_.3H_2_O (2.03 g, 8.28 mmol) was dissolved in ethanol (10 mL), and then amino‐2‐propanol (A2P) (1.24 g, 16.6 mmol) dissolved in ethanol (15 mL) was added. Upon addition the solution turned from clear pale blue to dark blue. The mixture was heated to reflux and stirred overnight. After 22 h, the solution was removed from heat, cooled, and filtered by using gravity, separating a blue solute from a blue precipitate. The solution was left at −5 °C to allow crystals to grow. After 12 days in the freezer, dark blue crystals suitable for X‐ray analysis had formed. Experimental analysis for [Cu(A2P)_2_(NO_3_)_2_]: Yield: 87 %; Elemental analysis found: C 22.82, H 5.77, N 16.39, calculated for [Cu(A2P)_2_(NO_3_)_2_] C 21.31, H 5.33, N 16.58; FTIR (cm^−1^): 3331 (N‐H), 3290 (N‐H), 3240 (N‐H), 3156 (N‐H), 2973 (C−H), 2943 (C−H), 1595 (N‐H bend). Mass spectrometry: ES‐LRMS [M]^+^ 337.0 (100 %).

### Plasma‐Assisted Inkjet Printing of Copper

#### Formulation Copper Inks

##### Formulation of Ink 1

Complex **1** (0.2301 g, 0.7 mmol, 0.14 m) was dissolved in deionized water (5 mL) and stirred for 20 min in air. The ink, which was a violet/blue color, was filtered through a 200 nm syringe filter before use, despite there being no visible precipitate.

##### Formulation of Ink 2

Complex **2** (0.2401 g, 0.7 mmol, 0.14 m) was dissolved in deionized water (5 mL) and stirred for 20 min in air. The ink, which was royal blue, was filtered through a 200 nm syringe filter before use, despite there being no visible precipitate.

##### Formulation of Ink 3

Complex **3** (0.2421 g, 0.7 mmol, 0.14 m) was dissolved in deionized water (5 mL) and stirred for 20 min in air. The ink, which was royal blue, was filtered through a 200 nm syringe filter before use, despite there being no visible precipitate.

#### Deposition of Conductive Copper

Inks **1**–**3** were all used to achieve coatings of copper in a semi‐industrial AP‐DBD reactor composed of two high‐voltage electrodes covered by a dielectric and a moving stage. Copper inks **1**–**3** were inkjet‐printed onto glass substrates by using a MicroMist ultrasonic nozzle operating at 120 kHz and coupled to an air‐shaping system from Sono‐Tek. The inkjet‐printing step was performed in the dynamic mode (100 mm s^−1^) and approximately (1 mL min^−1^) 83 μL cm^−2^ were deposited per cycle (three cycles of print/plasma in total). After printing, the coated substrate was exposed to an atmospheric‐pressure plasma discharge ignited in a mixture of argon and hydrogen (97.5:2.5), using a 10 000 Hz (2 W cm^−2^) sinusoidal signal generated by a Corona generator 7010R from SOFTAL electronic GmbH for 40 min. The discharge gap between the high‐voltage electrodes and the glass substrate was 1 mm.

## Conflict of interest

*The authors declare no conflict of interest*.
